# Acute exercise decreases PTP-1B protein level and improves insulin signaling in the liver of old rats

**DOI:** 10.1186/1742-4933-10-8

**Published:** 2013-02-25

**Authors:** Leandro Pereira de Moura, Luciana Santos Souza Pauli, Dennys Esper Cintra, Claudio Teodoro de Souza, Adelino Sanchez Ramos da Silva, Rodolfo Marinho, Maria Alice Rostom de Melo, Eduardo Rochete Ropelle, José Rodrigo Pauli

**Affiliations:** 1 Universidade Estadual Paulista, UNESP, Curso de Pós-Graduação em Ciências da Motricidade Humana, Rio Claro, SP, Brazil; 2 Faculdade de Ciências Aplicadas, Universidade Estadual de Campinas, Curso de Pós-Graduação em Nutrição, Esporte e Metabolismo. UNICAMP, Limeira, SP, Brazil; 3 Universidade do Extremo Sul Catarinense, Laboratório de Bioquímica e Fisiologia, Santa Catarina, Criciúma, SC, Brazil; 4 Universidade de São Paulo, Escola de Educação Física e Esporte, USP, Ribeirão Preto, SP, Brazil; 5 Curso de Ciências do Esporte, FCA-UNICAMP, Rua Pedro Zaccaria, 1300, Jardim Santa Luzia, Limeira, SP, Brazil

## Abstract

It is now commonly accepted that chronic inflammation associated with obesity during aging induces insulin resistance in the liver. In the present study, we investigated whether the improvement in insulin sensitivity and insulin signaling, mediated by acute exercise, could be associated with modulation of protein-tyrosine phosphatase 1B (PTP-1B) in the liver of old rats. Aging rats were subjected to swimming for two 1.5-h long bouts, separated by a 45 min rest period. Sixteen hours after the exercise, the rats were sacrificed and proteins from the insulin signaling pathway were analyzed by immunoblotting. Our results show that the fat mass was increased in old rats. The reduction in glucose disappearance rate (Kitt) observed in aged rats was restored 16 h after exercise. Aging increased the content of PTP-1B and attenuated insulin signaling in the liver of rats, a phenomenon that was reversed by exercise. Aging rats also increased the IRβ/PTP-1B and IRS-1/PTP-1B association in the liver when compared with young rats. Conversely, in the liver of exercised old rats, IRβ/PTP-1B and IRS-1/PTP-1B association was markedly decreased. Moreover, in the hepatic tissue of old rats, the insulin signalling was decreased and PEPCK and G6Pase levels were increased when compared with young rats. Interestingly, 16 h after acute exercise, the PEPCK and G6Pase protein level were decreased in the old exercised group. These results provide new insights into the mechanisms by which exercise restores insulin signalling in liver during aging.

## Introduction

Aging in both humans and rodents is associated with increased fasting and postprandial plasma insulin levels [1,2] and decreased in glucose tolerance [2,3] suggesting an insulin-resistant state. Dysregulation of hepatic glucose homeostasis in aging associated with obesity is mainly caused by increased gluconeogenesis. Suppression of hepatic glucose output has been shown to be an effective therapeutic approach for controlling serum high level glucose in type 2 diabetes. Insulin is an important hormone for suppressing liver gluconeogenesis mainly through Akt mediated phosphorylation and inactivation of Foxo1, a transcription factor that stimulates expression of gluconeogenic genes such as phosphoenolpyruvate carboxykinase and glucose-6-phosphatase (PEPCK and G6Pase) [4-6].

Great efforts have been directed to study the mechanism of insulin resistance in liver related with aging and obesity. In this scenario, protein tyrosine phosphatase 1B (PTP1B) has emerged as key phosphatase, induced by inflammation, that has been shown to be a negative regulator of the insulin signal transduction in insulin resistant states. PTP1B knockdown in rodents protects against diabetes and obesity, the two important metabolic diseases in modern society. Not surprisingly, PTP1B is a highly regarded target of the pharmaceutical industry in the treatment of these disorders [7].

PTP-1B can diminish or block insulin action by tyrosine dephosphorylation of the insulin receptor (IR), rendering it inactive, or by dephosphorylation of insulin receptor substrate 1 and 2 (IRS1/2) inhibiting their interactions with downstream signaling molecules in periphery and central nervous system [8-10]. Consistent with these studies, complete absence of PTP-1B in mice (PTP-1B−/−) results in increased systemic insulin sensitivity, improved glucose tolerance, and enhanced liver IR phosphorylation, establishing PTP-1B as a physiologically important IR and IRSs phosphatase [11,12].

Recent study showed at a molecular level that PTP-1B expression and enzymatic activity were up-regulated in liver of old mice [13]. In addition, Hirata and colleagues demonstrated that the increase in PTP1B protein level and/or association with IR in monosodium glutamate (MSG) treated-rats may contribute to the impaired insulin signaling mainly in liver and muscle [14]. 28-week-old-MSG rats presented an increase in IR/PTP1B interaction and a reduction in insulin signaling in liver, muscle and adipocytes, and a more pronounced insulin resistance [14]. Conversely, mice with liver-specific PTP-1B-deficiency improved insulin sensitivity [15,16].

These data implicate PTP-1B in the development of insulin resistance during aging and suggest that inhibition of this phosphatase might protect against age-dependent type 2 diabetes. In this context, physical exercise is known to be essential in the treatment of type 2 diabetes. The effects of physical exercise on glucose uptake and disposal have important implications for individuals with diabetes in terms of chronic metabolic control and the acute regulation of glucose homeostasis [17-19]. The molecular mechanisms associated with insulin sensitivity that are enhanced in response to exercise may be related to increased expression and/or the activation of key proteins that regulate glucose metabolism [20-22]. Several studies have demonstrated that exercise improves insulin signaling in hepatic tissue [5,6,23,24]; however, these effects of exercise have not yet been investigated in insulin resistance during aging.

In the present study, we investigated whether the improvement in insulin sensitivity and insulin signaling, mediated by acute exercise, could be associated with modulation of PTP-1B in the liver of old rats.

## Results

### Physiological and metabolic parameters

**Figure 1:**
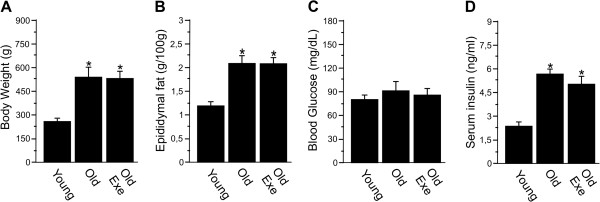
**Physiological and metabolic parameters of young, old (Old) and exercised old rats (Old Exe). A**, total body weight. **B**, epididymal fat weight. **C**. fasting glucose. **D**, fasting serum insulin. Bars represent means ± S.E.M. of six mice. *p <0.05 versus Young, ^#^p < 0.05 versus Old.

In Figure 1, comparative data regarding young rats (young), old sedentary rats (Old) and old rats submitted to an acute exercise protocol (Old Exe) are presented. Twenty-seven-month-old rats (Old and Old Exe) had a higher body weight and epididymal fat pad weight compared to young rats (Young). No significant variations were found in body weight and epididymal fat in Old Exe rats, after a single session of exercise, compared to Old rats (Figure 1A-1B). The fasting plasma glucose concentrations were similar between the groups; however, serum insulin was higher in old rats (Old and Old Exe), when compared with young rats (Figure 1C-1D).

### A single bout of exercise improves insulin signaling in the liver of old rats

**Figure 2:**
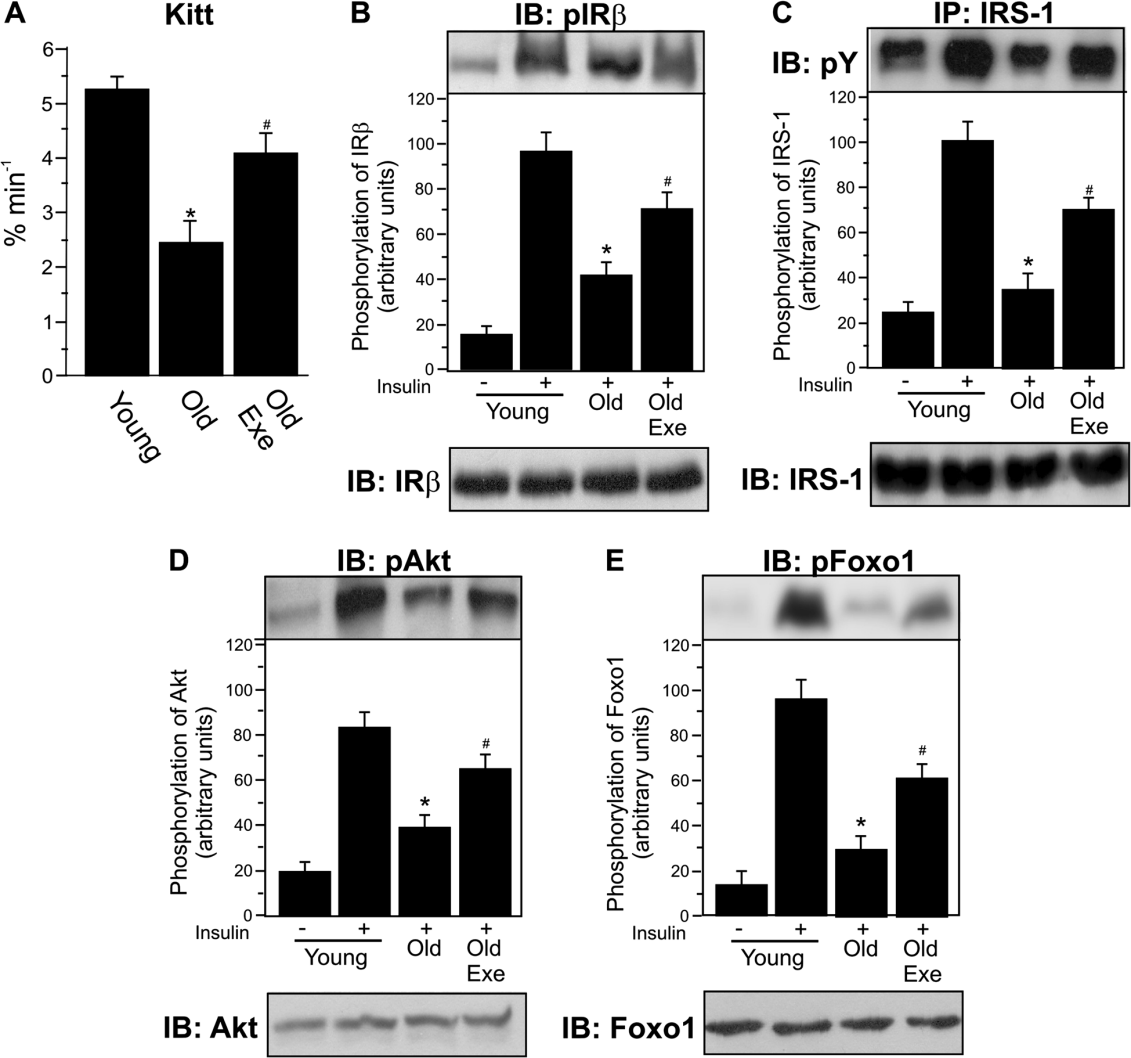
**Insulin signalling in liver of young, old (Old) and exercised old rats (Old Exe). A**. The decrease in the glucose disappearance rate (Kitt), induced by the aging associated with obesity, returned to the young rats level 16 h after acute exercise. Liver extracts from rats injected with saline or insulin were prepared as described in the Methods. **B**, tissue extracts were immunoblotting (IB) with anti-phospho-IRβ antibody (upper panel) or anti-IRβ antibody (lower panel). **C**, tissue extracts were also IP with anti-IRS-1 antibody and IB with anti-phosphotyrosine (pY) antibody (upper panels), and IB with anti-IRS-1 antibody (lower panel). **D**, liver extracts were IB with anti-phospho-Akt and anti-Akt antibody (upper and lower panel, respectively). **E**, liver extracts were IB with anti-phospho-Foxo1 and anti-Foxo1 antibody (upper and lower panel, respectively). The results of scanning densitometry were expressed as arbitrary units. Bars represent means ± S.E.M. of n = 6 rats. ^∗^P < 0.05, versus Young rats and ^#^p < 0.05 versus Old.

We observed increased insulin sensitivity 16 h after a single bout of exercise with old mice. We found significant impairment in the glucose disappearance rate (Kitt) in old mice at rest when compared with young mice. However, acute exercise restored the glucose disappearance rate in old exe mice (Figure 2A). In addition, insulin-induced increase in IRβ, IRS-1, Akt and Foxo1 phosphorylation in the liver of young mice, when compared to saline injection. In old group at rest, IRβ, IRS-1, Akt and Foxo1 phosphorylation were reduced after insulin injection when compared with young mice. Conversely, in the liver of the exercised mice, IRβ, IRS-1, Akt and Foxo1 phosphorylation increased compared with mice at rest (Figure 2B-E, upper parts, respectively). There was no difference in basal levels of IRβ, IRS-1, Akt and Foxo1 phosphorylation between the three groups (data not shown). Finally, the Foxo1 protein levels were not different between the groups (Figure 1B-E, lower parts).

### A single bout of exercise decreased the protein level of PTP-1B in aged rats

**Figure 3:**
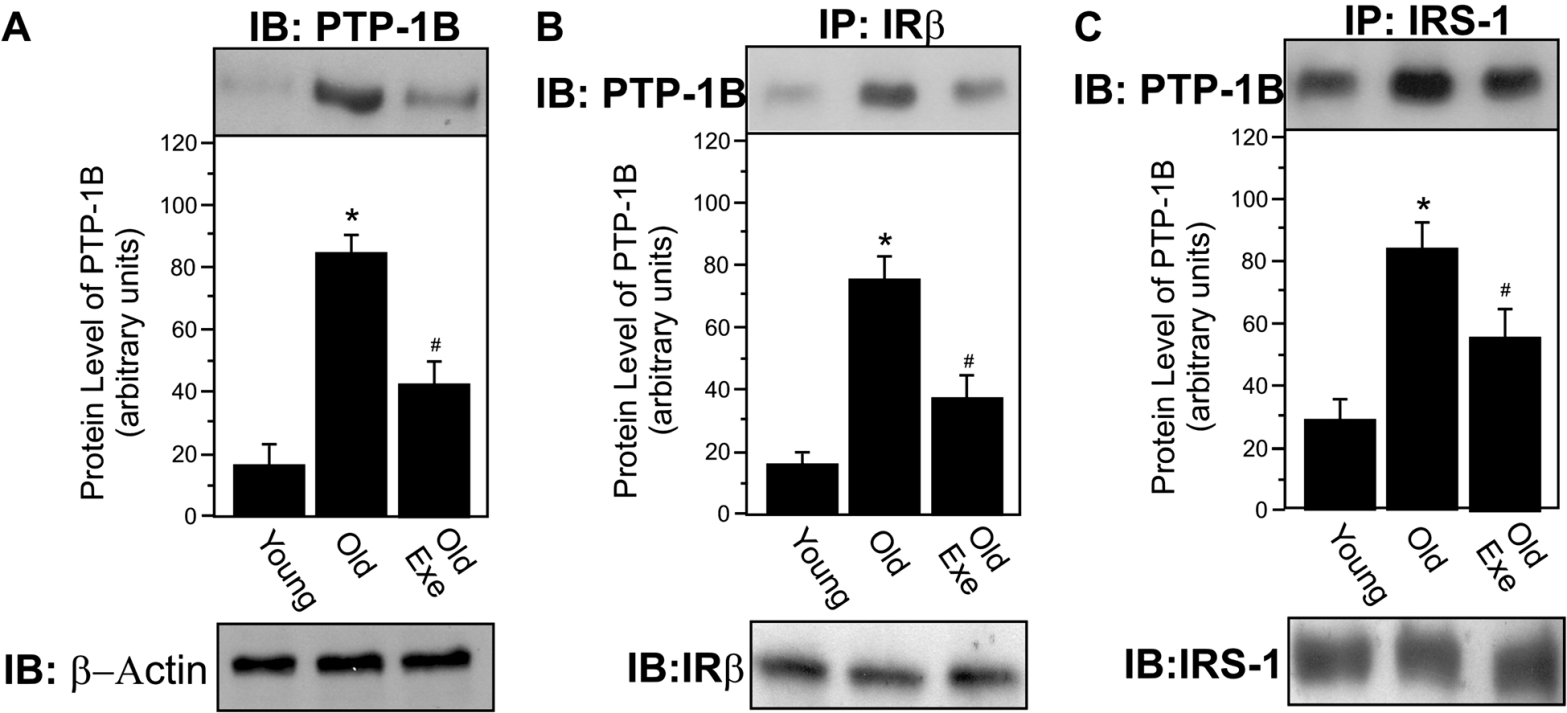
**Effect of acute exercise on PTP-1B protein levels and PTP1B association with IRβ and IRS-1. A**, PTP-1B protein level in Old and Old Exe rats were compared with Young group. **B**, tissue extracts were immunoprecipitated (IP) with anti-IRβ followed by immunoblotting (IB) with anti-PTP1B antibody or anti-IRβ antibody (upper and lower panels). **C**, IP with anti-IRS-1 followed by IB with anti-PTP1B antibody to evaluated the IRS-1–PTP1B association (upper panel). Liver extracts were also IP with anti-IRS-1 and IB with anti-IRS-1 antibody (lower panels). Immunoblot was performed employing anti-β-Actin antibody as the loaded protein (lower panels in **A**). The results of scanning densitometry were expressed as arbitrary units. Bars represent means ± S.E.M. of n = 6 rats. ^∗^P < 0.05, versus Young and ^#^P < 0.05, versus Old.

Aging increased the protein level of PTP-1B in the liver of old rats compared to control rats, a phenomenon that was reversed by acute exercise (Figure 3A). To further investigate the effect of acute exercise on this association in aged rats, we evaluated IRβ/PTP-1B and IRS-1/PTP-1B interaction in the liver of Old Exe rats. Aging rats increased the IRβ/PTP-1B and IRS-1/PTP-1B association in the liver when compared with young rats and. Conversely, in the liver of Old Exe rats, IRβ/PTP-1B and IRS-1/PTP-1B association was markedly decreased, when compared with Old rats (Figure 3B-C). The membrane was stripped and immunoblotted with anti-β-actin as loading protein (Figure 3A). Neither treatment changed β-actin protein levels.

### Acute exercise reduces PEPCK and G6Pase protein level in hepatic tissue of old rats

**Figure 4:**
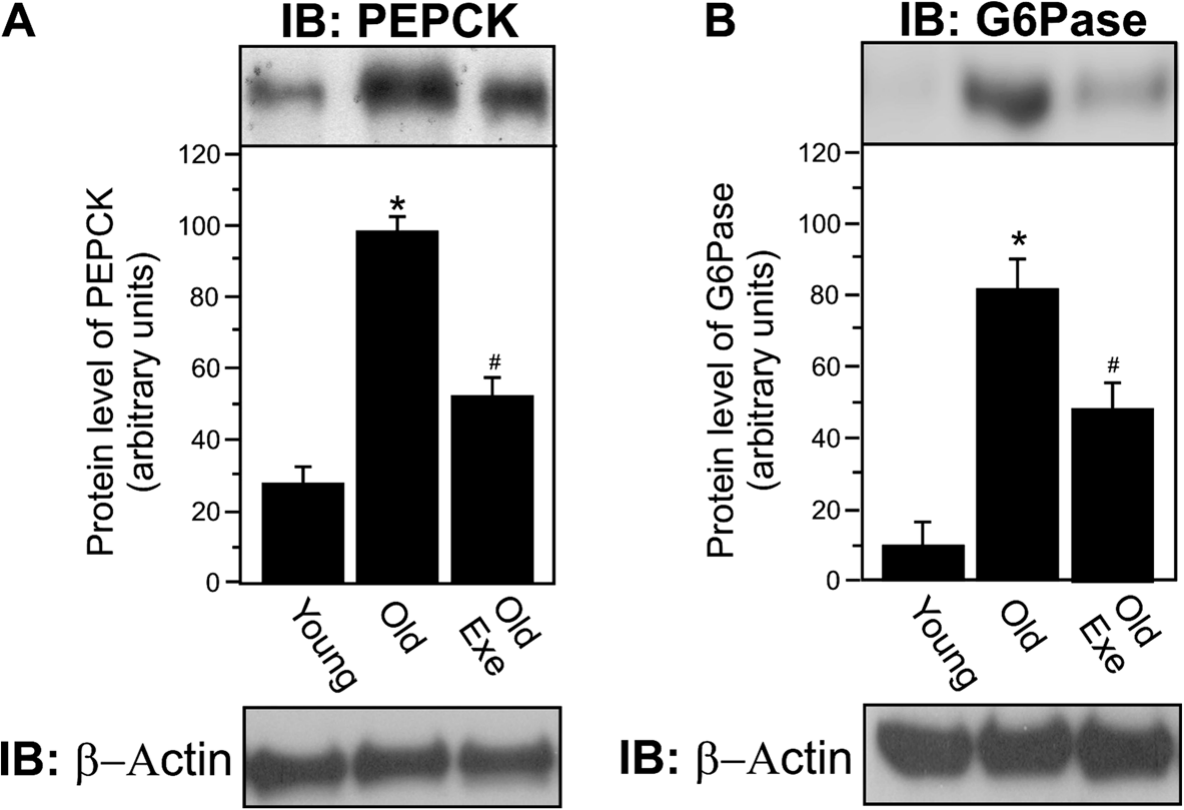
**Protein level of PEPCK and G6Pase in the liver of Young, Old and Old Exe groups. **Liver extract rats were submitted to immunobloting for PEPCK and G6Pase protein level, as described in Methods. **A**, tissue extracts were blotted (IB) with anti-PEPCK antibody. **B**, tissue extracts were blotted (IB) with anti-G6Pase antibody. Immunoblot was performed employing anti-β-Actin antibody as the loaded protein (lower panels in **A** and **B**). The results of scanning densitometry are expressed as arbitrary units. Bars represent means ± S.E.M. of n = 6 rats. ^∗^P < 0.05, Young rats and ^#^P < 0.05, versus Old.

We next observed the protein contents of PEPCK and G6Pase in the livers of the Young, Old and Old Exe groups under fasting conditions. In the hepatic tissue of Old rats at rest, the PEPCK and G6Pase levels were increased when compared with young rats (Figure 4A and B, respectively). Interestingly, 16 h after acute exercise, the PEPCK and G6Pase protein levels were decreased in the Old Exe group when compared with the Old group at rest (Figure 4A and B, respectively). The membrane was stripped and immunoblotted with anti-β-actin as loading protein (Figure 4A and 4B). Neither treatment changed β-actin protein levels.

## Discussion

The main finding of the present investigation is that aging is associated with the increased of PTP-1B protein level, resulting in the inhibition of insulin signaling by dephosphorylating of IR and IRS-1 in the hepatic tissue. On the other hand, it is known that genetic, pharmacological, and physiological PTP-1B inhibition increased IRβ tyrosine phosphorylation and insulin signaling in obese and old rodents [13,25-27]. Accordingly, it is recognized that the impairment of insulin action in liver is a hallmark feature of type 2 diabetes mellitus.

It is well established that insulin resistance associated with a progressive decrement in insulin action occurs with aging [25,28,29]. This decline has been attributed to chronological age itself and/or to a variety of secondary factors associated with the aging process, such as an increase in body fat and/or in central adiposity, and a reduction in spontaneous physical activity [28,30,31].

Correlational studies that employed more precise measurements of the confounding variables of body composition and fitness level found that age was responsible for little or none of the change in glucose tolerance occurring in the aged subjects [30-34]. Boden et al. found that age did not correlate with any parameter of glucose metabolism and that insulin sensitivity was determined more by body fat than age [33]. In 344 Fischer rats (animals that not become obese as do some rodent strains), insulin-stimulated glucose transport was reduced during maturation (2–8 mo) but was unchanged during aging (8–24 mo) [34].

In accordance, our results showed an increase in body weight in Wistar old rats, and this was accompanied by addition in adiposity (accumulation of epididymal adipose tissue) when compared with young rats. Taken together, these data suggest that the insulin resistance associated with aging may be caused, at least in part, by the obesity commonly found in aging. Therefore, the results of this study are important because they show that just one session of exercise is able to circumvent the negative effects of aging/obesity. These data are similar to the results of Ropelle and colleagues [5] that found that acute exercise can improve insulin sensitivity and insulin signaling in liver in the context of obesity. Corroborating with this data, Oliveira and colleagues [23] also showed that acute exercise improved insulin signaling in hepatic tissue of obese rats.

In addition, the old rats developed insulin resistance with impairment in the liver insulin signaling compared with the young rats. The old rats presented high levels of insulin, which is considered one of the main characteristics of obesity, with no change in glucose levels on fasting condition. The old rats seem to compensate the increased metabolic load and obesity-induced insulin resistance by the increase of insulin secretion from pancreas. Thus, these obese Wistar rats can maintain the glucose levels similar to the control animals. These results are in accordance with previous studies from our laboratory that used the same experimental model [25,28,35].

The ability of PTP-1B to negatively regulate insulin receptor kinase has been established at the molecular level [15] and the ablation of the PTP-1B gene yields mice displaying characteristics which suggest that inhibition of PTP-1B function may be an effective strategy for the treatment of diabetes and obesity during aging [11,13]. PTP-1B constitutes a family of phosphatases, including PTP-1B, SHP1, SHP2, and LAR, which act to reverse tyrosine kinase action [36]. PTP-1B is a major PTP implicated in the regulation of insulin action, including in the insulin-resistant state [11,37]. PTP-1B-deficient mice are more sensitive to insulin and are more resistant to diet-induced obesity than wild-type mice [10]. Diabetic mice treated intraperitoneally with PTP-1B antisense oligonucleotides have lower PTP-1B protein levels in liver, leading to decreases in fat, plasma insulin, and blood glucose levels [38]. These findings indicate that inhibition or downregulation of PTP-1B is an effective strategy for improving insulin sensitivity. In accordance, our results show decreased activity and protein content of PTP-1B in old rats after a single bout of exercise. Furthermore, the reduction of PTP-1B activity in rats submitted to acute exercise was accompanied by increased insulin signalling and correlates with increases in tyrosyl phosphorylation of IR and IRS-1 and with reduction of IR/PTP-1B and IRS-1/PTP-1B association in liver.

Downstream of IR and IRS-1, Foxo1 is an important regulator that modulates the expression of gluconeogenic genes in the nucleus, and this is mediated by Akt phosphorylation [5,6]. Low levels of Akt and Foxo1 phosphorylation were found in the livers of obese mice [5]. In accordance with these results, we observed that aging associated with weight gain and adiposity reduced Akt activity and Foxo1 phosphorylation, contributing to fasting hyperglycemia. Interestingly, acute exercise increased Akt and Foxo1 phosphorylation, reducing the fasting glucose levels. Our results regarding the improvement in insulin signaling, mediated by exercise in the hepatic tissue, were also observed in previous studies [5,22,39].

Once phosphorylated, Foxo1 translocates to the cytoplasm in response to insulin and reduces gluconeogenic gene transcription. In a previous study, Puigserver and colleagues [4] showed that Foxo1 and PGC-1α can physically interact with each other and that the combined action of PGC-1α and Foxo1 in various liver cell types results in a synergistic induction of endogenous G6Pase gene expression. Thus, PGC-1α stimulates G6Pase gene expression, in part, through a direct interaction with Foxo1 bound to the G6Pase promoter. We observed high levels of PEPCK and G6Pase in the liver of old rats. These data are in accordance with several studies that analyzed mice with severe insulin resistance [5,6,40].

Moreover, our data provide evidence that a single bout of exercise improves insulin signaling, increases the basal levels of Foxo1 phosphorylation, leading to a reduction in PEPCK and G6Pase protein contents in old rats. The activation of the insulin signaling pathway in liver and the reduction in gluconeogenic enzymes activities (PEPCK and G6Pase) culminate in a rapid reduction in hepatic glucose production [5,6]. In contrast, when hepatic insulin signaling is impaired, the suppression of gluconeogenic pathways is inadequate, leading to elevated levels of glucose and insulin responses during postprandial and fasting conditions [5,6].

In this study, we did not evaluate PGC1α protein level, PGC1α/Foxo1 association and the hepatic glucose production. The measurement of insulin sensitivity through insulin tolerance test is a whole body measurement; however, the insulin signaling was enhanced in liver. Thus, one limitation of our study is that we do not provide any evidence that hepatic glucose output was decreased in liver. However, in previous investigation, we have shown that the reduction in Foxo1 and PGC-1α content using different approaches diminished hepatic glucose production, as evaluated by hyperinsulinaemic-euglycaemic clamp procedures [5]. The reduction in gluconeogenic gene levels observed in exercised old animals is in accordance with Heled and co-authors, which showed that physical exercise enhances hepatic insulin signaling and inhibits PEPCK activity in diabetes-prone Psammomys obese [22]. Our results show a decreased protein content of PTP-1B, 16 h after acute exercise in old rats in parallel with an increase in IR autophosphorylation, and IRS-1 phosphorylation, which certainly contribute to improved insulin sensitivity.

The molecular mechanisms that account for this effect of physical activity on decreased expression PTP-1B are not completely known. Recently, Sun et al. show that SIRT1 is downregulated in insulin-resistant cells and tissues and that knockdown or inhibition of SIRT1 induces insulin resistance [41]. Furthermore, increased expression of SIRT1 improved insulin sensitivity, especially under insulin-resistant conditions. Similarly, resveratrol, a SIRT1 activator, enhanced insulin sensitivity in vitro in a SIRT1-dependent manner and attenuated high-fat-diet-induced insulin resistance in vivo. It is well established that the effect of SIRT1 on insulin resistance is mediated by the repression of PTP1B transcription at the chromatin level [41]. The improvement of insulin sensitivity by SIRT1 has implications in the treatment of insulin resistance and type 2 diabetes.

In previous study, exercised old rats show increased in SIRT1 expression in the skeletal muscle when compared with old rats at rest [25]. Thus, it is possible that this reduction in PTP1B in exercised rats may be mediated, at least in part, by an increase in SIRT1. Since exercise increase expression SIRT1 [42,43], we believe that other beneficial effects of exercise may also be mediated by this sirtuin. These data are important since inflammation inhibition in the liver represents a potential target therapy to combat the insulin resistance and development of nonalcoholic fatty liver disease.

Other data suggest that PTP1B overexpression in multiple tissues in obesity is regulated by inflammation. Zabolotny and colleagues determined that tumor necrosis factor alpha (TNFα) administration increased PTP1B mRNA levels in skeletal muscle, adipose tissue, liver and hypothalami of mice [44]. Likewise, it was demonstrated that TNFα-induced recruitment of NFkappaB (NF-κB) subunit p65 to the PTP1B promoter in vitro and in vivo [44]. On the other hand, we demonstrated that acute exercise improved insulin sensitivity in the skeletal muscle of obese [45,46] and aged animals [25] by reducing I-kappa-B Kinase-beta (IKKβ) signaling and PTP1B activity, suggesting that physical exercise suppress the PTP1B expression through the anti-inflammatory mechanism in several tissues.

In conclusion, our data demonstrate that acute exercise improves insulin sensitivity in the liver. The effect of exercise on insulin action is further supported by our findings that exercised rats show an increased the transcription factor phosphorylation in old rats, a mechanism by which exercise may diminish the content of the glyconeogenic enzymes, PEPCK and G6Pase, and consequently hepatic glucose production. These data provide considerable progress in our understanding of the molecular events that link physical exercise to an improvement insulin signaling in liver and fasting hyperglycemia during aging.

## Materials and methods

### Animals

The experimental procedures involving rats were performed in accordance with the guidelines of the Brazilian College for Animal Experimentation and were approved by the ethics committee at the State University of Campinas. Six rats (n = 6) were used per group (young group: rats of 3 months of age), old sedentary rats (group Old: 21 months of age), and exercised 21-month-old rats (group Old Exe).

### Exercise protocol

Rats were adapted to swimming for 10 min during 3 days. The animals swam in groups of three in plastic barrels of 45 cm in diameter that were filled to a depth of 60 cm, for two 1.5-h long bouts, separated by a 45-min rest period and the water temperature was maintained at∽ 32°C. This exercise protocol was adapted from a previously published procedure [24]. After the last bout of exercise, animals were fed *ad libitum* for 10 h and food was withdrawn 6 h before the tissue extraction, with free access to water, totalizing 16 hours of recovery. The rats used in the experiment were anesthetized with an intraperitoneal (i.p) injection of ketamine chlorohydrate (50 mg/kg; Ketalar; Parke-Davis, Ann Arbor, MI) and xylazine (20 mg/kg; Rompun; Bayer, Leverkusen), and decapitated.

### Insulin tolerance test (ITT), serum glucose and insulin quantification

The ITT was realized 16 h after the exercise protocol. Briefly, 1.5 UI/kg of human recombinant insulin (Humulin R) from Eli Lilly (Indianapolis, IN, USA) was injected intraperitoneally in anesthetized rats, the blood samples were collected at 0, 5, 10, 15, 20, 25 and 30 min from the tail for serum glucose determination. The rate constant for plasma glucose disappearance (Kitt) was calculated using the formula 0.693/(t1/2). The plasma glucose t1/2 was calculated from the slope of last square analysis of the plasma glucose concentration during the linear phase of declive [47]. The plasma glucose level was determined by a colorimetric method using a glucosemeter (Advantage. Boehringer Mannheim, USA). Plasma was separated by centrifugation (1100 x g) for 15 min at 4°C and stored at∽ 80°C until assayed. Serum insulin was determined using a commercially available Enzyme Linked Immunosorbent Assay (ELISA) kit (Crystal Chem Inc., Chicago, IL).

### Protein analysis by immunoprecipitation and immunoblotting

For tissue collection, the abdominal cavity was opened, the portal vein exposed, and 0.2 ml of normal saline with or without insulin (10^-6^ mol/L) was injected. After the insulin injection, hepatic tissue fragments were excised. The tissues were ablated, pooled, minced coarsely and homogenized immediately in extraction buffer (1% Triton-X 100, 100 mM Tris, Ph 7.4, containing 100 mM sodium pyrophosphate, 100 mM sodium fluoride, 10 mM EDTA, 10 mM sodium vanadate, 2 mM PMSF and 0.1 mg of aprotinin/mL) at 4°C with a Polytron PTA 20S generator (Brinkmann Instruments model PT 10/35) operated at maximum speed for 30 s. The extracts were centrifuged at 9000 x g and 4°C in a Beckman 70.1 Ti rotor (Palo Alto, CA) for 40 min to remove insoluble material, and the supernatants of these tissues were used for protein quantification, performed by the Bradford method [48]. Equal amounts of protein were used for immunoprecipitation with 10 ml of the following antibodies: anti-IRβ and anti-IRS-1 (Santa Cruz Biotechnology; Santa Cruz, California, USA), as indicated.

The immunomplex were precipitated with protein A-Sepharose 6 MB (Pharmacia; Uppsala, Sweden) and then washed three times with 50 mM Tris (pH 7.4) containing 2 mM sodium vanadate, and 0.1% Triton X-100. After this procedure, proteins were denatured by boiling in Laemmli sample buffer containing 100 mM DTT, run on SDS-PAGE, transferred to nitrocellulose membranes, which were blocked, probed and developed as described previously [49].

Antibodies used for immunoblotting were anti-phosphotyrosine, anti-phospho-IRβ, anti-IRβ, anti-IRS-1, anti-phospho [Ser473] Akt, anti-Akt, anti-PEPCK, anti-G6pase, anti-beta-Actin (Santa Cruz Biotechnology Inc., CA, USA) anti-phospho-Foxo1, anti-Foxo1 (Cell Signaling Technology, MA, USA), anti-PTP-1B (Upstate Biotechnology, NY, USA). Blots were exposed to pre-flashed Kodak XAR film with Cronex Lightning Plus intensifying screens at 80°C for 12–48 h. Band intensities were quantified by optical desitometry (Scion Image software, ScionCorp, Frederick, MD).

### Statistical analysis

Results are expressed as mean ± standard error of the mean (SEM). Differences between the groups were evaluated using one-way analysis of variance (ANOVA). When ANOVA indicated significance, a Bonferroni post hoc test was performed. A probability of less than 0.05 was considered significant. The software used for analysis of the data was the Statistical Package for the Social Sciences (SPSS) version 17.0 for Windows

## Competing interest

The authors declare that there are no conflicts of interest regarding the present study.

## Authors’ contributions

LPM, LSSP and JRP had the overall responsibilities of the experiment design, data collect and statistical analysis, and wrote the manuscript. DEC, CTS, ASRS, RM and MARM carried out the data analyses and wrote the discussion of the article. All the authors have read and approved the final manuscript.
